# Linezolid Dependence in *Staphylococcus epidermidis* Bloodstream Isolates

**DOI:** 10.3201/eid1901.111527

**Published:** 2013-01

**Authors:** Spyros Pournaras, Eleni Ntokou, Olympia Zarkotou, Kyriaki Ranellou, Katerina Themeli-Digalaki, Constantinos Stathopoulos, Athanassios Tsakris

**Affiliations:** Author affiliations: University of Thessaly Medical School, Larissa, Greece (S. Pournaras, E. Ntokou);; Tzaneio General Hospital, Piraeus, Greece (O. Zarkotou, K. Themeli-Digalaki);; University of Athens Medical School, Athens, Greece (K. Ranellou, A. Tsakris);; University of Patras School of Medicine, Patras, Greece (C. Stathopoulos)

**Keywords:** L3 mutations, 23S rDNA mutations, growth rate, protein synthesis, ribosome, Staphylococcus epidermidis, bacteria, antibiotic, antimicrobial resistance

## Abstract

We document linezolid dependence among 5 highly linezolid-resistant (LRSE) *Staphylococcus epidermidis* bloodstream isolates that grew substantially faster at 32 µg/mL linezolid presence. These isolates carried the mutations T2504A and C2534T in multiple 23S rRNA copies and 2 mutations leading to relevant amino acid substitutions in L3 protein. Linezolid dependence could account for increasing LRSE emergence.

Linezolid is highly effective against *Staphylococcus epidermidis* (*1*). Linezolid-resistant *S. epidermidis* (LRSE) isolates are limited worldwide (*2*), and few LRSE outbreaks have occurred (*3*,*4*). Linezolid resistance in *S. epidermidis* has been attributed to specific 23S rRNA mutations (G2576U, G2447U, U2504A, C2534U, and G2631U) (*5*,*6*), *cfr* gene (*7*), or mutations in ribosomal proteins L3, L4, and L22 (*7*).

Dependence on linezolid for bacterial growth has not been reported but has been described for other antimicrobial drugs (*8*–*10*). We report the characteristics of partially linezolid-dependent LRSE causing bloodstream infections (BSIs).

## The Study

Twenty-seven LRSE isolates were randomly selected for study among the 46 single-patient LRSE isolates recovered from BSIs in Tzaneio General Hospital (Piraeus, Greece) during 2008–2010. Isolates were identified by Vitek 2 (bioMérieux, Marcy l’Etoile, France). Chloramphenicol and clindamycin MIC was determined by E-test (bioMerieux) and linezolid MIC by using broth microdilution (*11*).

The 27 LRSE isolates were tested by pulsed-field gel electrophoresis (PFGE) as described (*12*) and screened for *cfr* gene (*7*). Mutations in the peptidyl-transferase center were identified for each separate 23S rRNA copy as described (*13*).

In 8 LRSE isolates representing all PFGE types, genes encoding the L3, L4, and L22 ribosomal proteins that factor in ribosome assembly were sequenced to identify mutations conferring linezolid resistance (*6*). Nucleotide and amino acid sequences were analyzed by using Lasergene software (DNASTAR, Madison, WI, USA) and compared with those of the linezolid-susceptible *S. epidermidis* (LSSE) strain ATCC12228 (GenBank accession no. AE015929).

Growth curves were conducted in the presence and absence of linezolid for the above 8 LRSE isolates, 1 clinical LSSE isolate (A1521, linezolid MIC 2 μg/mL), and the ATCC 29213 *S. aureus* strain (linezolid MIC 0.5 μg/mL) as controls. Linezolid concentrations tested were half-MIC for controls and 3 LRSE isolates with low MIC (16–32 μg/mL) and 8, 16, 32, 64, and 128 μg/mL for 5 LRSE isolates with MIC >256 μg/mL. Growth curves were performed in triplicate by diluting 20 μL Mueller-Hinton broth culture in 2 mL broth, followed by incubation at 37°C under constant shaking; turbidity of cultures (McFarland scale) was measured every 6 h for 36 h. We statistically compared isolate growth at each time point using the paired *t* test and Minitab software version 13.31 (www.minitab. com); p<0.05 indicated statistical significance.

We retrospectively examined medical records (anonymized demographic data, clinical characteristics, comorbidities, prior linezolid treatment for >3 days, and in-hospital deaths) of the 27 patients harboring LRSE to ascertain factors influencing resistance acquisition and outbreak persistence. Each of the 27 patients yielding LRSE had prolonged hospitalization and carried a central venous catheter. Twenty-one were mechanically ventilated, and 25 received linezolid treatment (Table 1).

**Table 1:** Demographic and clinical characteristics of 27 patients with bloodstream infections who yielded linezolid-resistant *Staphylococcus epidermidis*, Greece, 2008–2010

Patient characteristics	Finding
Mean age, y, ± SD	46.9 ± 21.7
Male sex, no. (%)	16 (59.3)
Comorbidities >2, no. (%)	8 (29.6)
Mean hospital stay, d ± SD	27.1 ± 9.8
Use of mechanical ventilation	
Isolates recovered during ventilation, no. (%)	21 (77.8)
Mean duration, d ± SD	23.4 ± 9.7
Presence of central venous catheter	
No. (%) patients	27 (100)
Mean duration, d ± SD	27.1 ± 9.8
Presence of foreign material, no. (%)	11 (40.7)
Admission from other hospital, no. (%)	6 (22.2)
Prior hospitalization, no. (%)	10 (37.0)
Linezolid administration	
No. (%) patients	25 (92.6)
Mean duration, d ± SD	12.9 ± 7.4
In-hospital deaths, %	18.5

Linezolid MICs were >256 μg/mL for 23 LRSE isolates and 8–32 μg/mL for 4 LRSE isolates. All isolates were co-resistant to clindamycin and chloramphenicol, but the *cfr* gene was not detected by PCR in any isolate (*7*). Three PFGE types were identified. PFGE type I comprised the 23 highly LRSE isolates, which all carried mutations T2504A and C2534T; 3 LRSE isolates were related to each other (type II) and carried the mutations G2576T and C2534T; and 1 LRSE isolate was unique (type III) and carried G2576T along with novel mutations C2356T or T2334C in different 23S rRNA copies each. All isolates had mutations in 3–6 copies of 23S rRNA. The *cfr* gene was not detected in any isolate.

**Figure 1:**
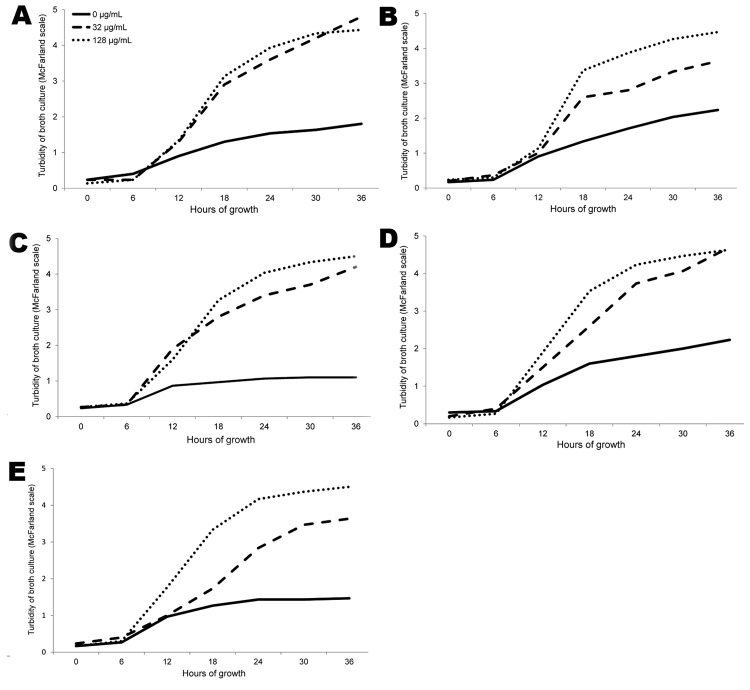
LRSE isolated from patients with bloodstream infections, Greece, 2008–2010. Effect of growth under exposure to linezolid at 128 μg/mL is shown for the 5 highly LRSE: A) A2562[1], B) E371, C) A2864, D) 217, and E) 605–2. LRSE, linezolid-resistant *Staphylococcus epidermidis.*

**Figure 2:**
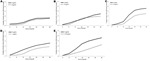
LRSE isolated from patients with bloodstream infections, Greece, 2008–2010. Effect of growth under exposure to linezolid at half-MIC is shown for the 3 low-level LRSE: A) A2570, B) A1702, and C) A2490; and at half-MIC for the 2 linezolid-susceptible control isolates: D) A1521 and E) ATCC 29213. LRSE, linezolid-resistant *Staphylococcus epidermidis*.

Characteristics of the 8 LRSE isolates tested by growth analysis are shown in Table 2; curves of the 5 highly LRSE isolates at 0, 32, and 128 μg/mL linezolid and of the 3 low-level LRSE and controls at half-MIC linezolid are shown in Figures 1 and 2. The growth of all 8 LRSE isolates was significantly slower than for the *S. aureus* control (p<0.05 at 24 h and at 36 h incubation for all isolates). Exposure to 8 μg/mL linezolid did not affect growth of the 5 highly LRSE isolates (p>0.05 for all isolates; data not shown). The 3 low-level LRSE isolates and the LSSE control showed moderately slower growth (p>0.05 at 24 h and 36 h) and the *S. aureus* control showed significantly slower growth (p<0.05 at 24 h and 36 h) at half-MIC linezolid than without linezolid. However, exposure of the 5 highly LRSE isolates to 32 and 128 μg/mL linezolid resulted in significantly faster growth compared with linezolid absence (p<0.05 at 24 and 36 h with 32 μg/mL linezolid and p<0.01 at 24 and 36 h with 128 μg/mL linezolid for all 5 isolates), suggesting partial linezolid dependence. Remarkably, all 5 linezolid-dependent LRSE isolates grew significantly faster with 128 μg/mL linezolid than did the 3 low-level LRSE isolates and the LSSE control with half-MIC and without linezolid (p<0.05 at 24 h and 36 h). Furthermore, 3 linezolid-dependent LRSE isolates (A2864, A2562[1], 217) grew significantly faster with 128 μg/mL linezolid than did the *S. aureus* control without linezolid (p<0.05 at 24 h and 36 h).

**Table 2:** Characteristics of 8 linezolid-resistant *Staphylococcus epidermidis* isolates tested for growth in the presence and absence of linezolid, Greece, 2008–2010*

Isolate designation	PFGE type	Mutations in each allele of the 23S rRNA							MIC, μg/mL		
		*rrlA*	*rrlB*	*rrlC*	*rrlD*	*rrlE*	*rrlF*		Linezolid	Chloramphenicol	Clindamycin
A2562(1)	I	T2504A	–	T2504A	T2504A	T2504A	T2504A		>256	>256	>256
		C2534T	–	C2534T	C2534T	C2534T	C2534T				
A2570	II	–	–	–	C2534T	C2534T	C2534T		16	>256	>256
		–	–	–	G2576T	G2576T	G2576T				
E371	I	T2504A	–	T2504A	T2504A	T2504A	T2504A		>256	>256	>256
		C2534T	–	C2534T	C2534T	C2534T	C2534T				
A2864	I	T2504A	–	T2504A	T2504A	T2504A	T2504A		>256	>256	>256
		C2534T	–	–	C2534T	C2534T	C2534T				
217	I	C2534T	T2504A	–	T2504A	T2504A	T2504A		>256	>256	>256
		–	C2534T	–	C2534T	–	C2534T				
605-2	I	T2504A	–	–	T2504A	T2504A	T2504A		>256	>256	>256
		–	–	–	C2534T	C2534T	C2534T				
A1702	II	G2576T	G2576T	G2576T	C2534T	G2576T	C2534T		32	64	>256
		–	–	–	G2576T	–	G2576T				
A2490	III	C2356T	T2334C	–	C2356T	C2356T	C2356T		32	>256	>256
		G2576T	G2576T	–	G2576T	G2576T	G2576T				

*PFGE, pulsed-field gel electrophoresis; –, absence of mutated position in the respective 23S rRNA copy.

The 5 linezolid-dependent LRSE isolates had 2 potentially relevant amino acid substitutions, G152D (shift from a small amino acid to a negative hydrophilic) and D159Y (shift of hydrophilic to hydrophobic amino acid), and a less significant one (L101V) in L3 protein. No amino acid changes were observed in the remaining 3 isolates tested for proteins L3, L4, and L22 or in proteins L4 and L22 for any isolate tested.

## Conclusions

All study isolates were recovered from patients with BSIs, indicating relatively high infectivity. Most of the LRSE isolates were clonally related, but 3 distinct PFGE types were detected, implying that linezolid resistance emerged in at least 3 different strains, which subsequently spread between patients. However, all linezolid-dependent isolates were clonal, implying that dependence possibly emerged once or on few occasions.

Antimicrobial drug resistance associated with dependence has been described for streptomycin and vancomycin (*8*–*10*). While investigating linezolid resistance in 8 LRSE isolates, we observed slower growth without linezolid than in controls, possibly resulting from mutations conferring resistance-exerted fitness cost. Surprisingly, linezolid concentrations at >32 μg/mL caused impressive growth acceleration in all 5 highly LRSE isolates, rendering significantly faster growth than without linezolid. Linezolid dependence is evident starting from relatively low linezolid concentrations, against which LRSE may be exposed in vivo during linezolid treatment. In fact, most of these 27 patients, including all 5 harboring linezolid-dependent LRSE, had prolonged linezolid treatment before yielding LRSE. This exposure also may have fostered the transition from resistance to dependence as suggested previously in vancomycin-dependent enterococci (*8*). Therefore, the high intrahospital linezolid consumption may favor not only LRSE selection but also their competitive survival. Should linezolid dependence prove common in highly LRSE isolates, it could explain their increasing clinical occurrence and the emergence of LRSE outbreaks (*3*,*4*,*13*). To support this hypothesis, growth with and without linezolid needs to be tested on larger collections of LRSE isolates. Growth characteristics of LRSE isolates reported previously should also be studied.

The underlying mechanism by which linezolid binding to the mutated ribosomal subunits enhances growth may be complex. All 5 linezolid-dependent isolates harbored mutations T2504A combined with C2534T, whereas the linezolid-nondependent isolates harbored other mutations in 23S rRNA genes (Table 2). Also, only the linezolid-dependent isolates carried mutations in the ribosomal protein L3, known to stimulate ribosome assembly. The coupling of rRNA synthesis from precursor RNA molecules and ribosome assembly possibly affects the overall rate of protein synthesis in vivo (*14*). Linezolid may interfere in this interaction, thus affecting the ribosomal assembly and enabling interactions with precursor forms of the 50S subunit, as demonstrated for erythromycin (*15*). We speculate that linezolid-dependent cells may possess linezolid-dependent ribosomal precursor particles exhibiting different structural conformation, which favors a faster rate of the overall protein synthesis recovery. This feature might explain the linezolid-dependent growth of the isolated strains. Further functional ribosomal characterization is required to elucidate linezolid dependence.
